# The application value of metagenomic next-generation sequencing in community-acquired purulent meningitis after antibiotic intervention

**DOI:** 10.1186/s12879-023-08672-4

**Published:** 2023-10-12

**Authors:** Lijuan Shangguan, Lanping Xue, Jing Shang, Hailong Wang

**Affiliations:** 1 Department of Neurology, Shanxi Bethune Hospital, Shanxi Academy of Medical Sciences, Tongji Shanxi Hospital, Third Hospital of Shanxi Medical University, Taiyuan, 030032 China; 2 Tongji Hospital, Tongji Medical College, Huazhong University of Science and Technology, Wuhan, 430030 China

**Keywords:** Metagenomic next-generation sequencing (mNGS), Community-acquired purulent meningitis, Antibiotic, Detection rate

## Abstract

**Background:**

Bacteria account for nearly one third of the causes of community-acquired central nervous system infections, and traditional diagnostic methods are based on culture results, which are time-consuming and have a low detection rate leading to delayed diagnosis and treatment. Since metagenomic next-generation sequencing (mNGS) has the advantages of high timeliness and only detecting microbial trace gene fragments, it has been used more widely in recent years. Based on this, we explored whether the application of cerebrospinal fluid (CSF) mNGS is advantageous in patients with community-acquired purulent meningitis, especially in people who have already used antibiotics.

**Methods:**

This was a retrospective study of 63 patients with community-acquired purulent meningitis admitted to the Department of Neurology of Shanxi Bethune Hospital from March 2018 to November 2022. Data were systematically collected and classified into CSF culture group, blood culture group and CSF mNGS group according to different detection methods, and the total detection rate of each method was calculated. Each group of patients was divided into two subgroups according to whether antibiotics were used before sampling. The detection rates of the three groups were compared within and between groups to explore whether mNGS has advantages over traditional methods and the influence of antibiotic use on detection rates of the three methods.

**Results:**

Among the 63 patients, the cases of CSF culture, blood culture and CSF mNGS were 56, 46, 44, respectively. The total detection rates of the three methods were 17.86%, 36.96%, 81.82%, with statistical differences (*p* < 0.05),suggesting that the detection rate of mNGS was higher than CSF culture (*p* < 0.05) and blood culture (*p* < 0.05),and the detection rate of blood culture higher than CSF culture (*p* < 0.05). Further grouping found that without antibiotics, the detection rates of CSF culture, blood culture and CSF mNGS were 28.57%, 56.25% and 88.89%, with statistical differences (*p* < 0.05), and the detection rate of CSF mNGS was higher than that of CSF culture (*p* < 0.05), but there was no statistical difference between CSF and blood culture (*p* > 0.05), nor between blood culture and CSF mNGS (*p* > 0.05). The detection rates of the three groups with antibiotics were 14.29%, 26.67% and 80.00%, with statistical differences (*p* < 0.05), and the detection rate of CSF mNGS was still higher than CSF culture (*p* < 0.05) and blood culture (*p* < 0.05). However, the detection rate of CSF mNGS also decreased after antibiotics were used for more than 3 days.

**Conclusions:**

The detection rate of CSF mNGS in patients with purulent meningitis is higher than traditional methods, especially in patients who have been given antibiotics, but the detection rate will decrease with the extension of antibiotic use.

## Background

Infectious diseases are one of the leading causes of morbidity and mortality worldwide [1]. As a common infectious disease of the central nervous system (CNS), purulent meningitis has a very high disability rate and mortality rate due to the rapid propagation of pathogenic bacteria and the characteristics of being difficult to detect. At present, the gold standard for the diagnosis of purulent meningitis is still CSF culture, which has a low positive rate. Previous studies [2] have shown that sending CSF samples for inspection several times after repeated lumbar puncture can improve the detection rate. However, the increase of repeated lumbar puncture will increase the pain and economic burden of patients, and the pathogen detection rate decreases with the extension of empirical antibiotic use [3]. Therefore, how to quickly and efficiently identify the pathogenic bacteria has been the difficulty in the diagnosis and treatment of this disease. In recent years, the emerging metagenomic next-generation sequencing(mNGS) technology is highly regarded in the diagnosis of CNS infections because it can identify pathogenic pathogens only by identifying tiny gene fragments of pathogenic microorganisms. Several relevant case reports and clinical studies have emerged one after another [4–8]. In April 2022, Chinese scholars also published a consensus on related applications. As purulent meningitis is more common in children, most studies on the detection of mNGS in patients treated with antibiotics have focused on this group [9, 10]. There are few studies in adults, and most of them focus on the use of mNGS in the entire spectrum of infectious diseases of the CNS [11, 12], and no further discussion on purulent meningitis has been conducted. This study retrospectively analyzed the detection of community-acquired purulent meningitis pathogens by CSF mNGS in the real world, with the aim to explore whether this method is superior to traditional methods in adult patients after antibiotic intervention.

## Methods

### Study population and sample collection

**Fig. 1:**
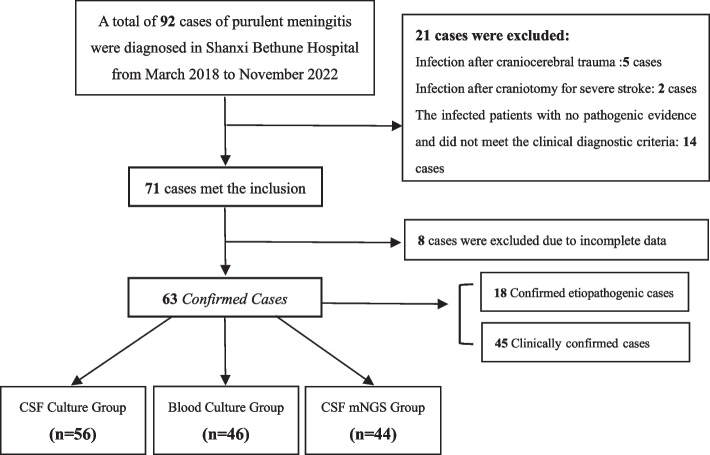
Flow chart

A total of 92 patients diagnosed with purulent meningitis/meningoencephalitis admitted to the Department of Neurology, Bethune Hospital, Shanxi Province from March 2018 to November 2022 since mNGS has been widely used in clinical practice in our hospital. According to Flow chart (Fig. 1), 63 confirmed cases were finally enrolled. All patient data were collected including clinical manifestations, physical examination, craniocerebral imaging, blood cell count, CRP, PCT, CSF cell count, CSF protein and glucose, focusing on blood culture, CSF culture, CSF mNGS results and antibiotic use, and the above contents were summarized.

This study was reviewed and approved by the Medical Ethics Committee of the Shanxi Bethune Hospital in Shanxi, China. (No. YXLL-2023–102). Informed consent was waived because this was a retrospective study. We obtained patient data from the Medical Records and Statistics Room. We anonymously analyzed the data. The use of raw data was permitted by the Medical Ethics Committee of Shanxi Bethune Hospital.

*Inclusion criteria* (must meet 1, 2, and simultaneously meet 3 or 4): 1. Patients present with clinical manifestations or signs of meningitis or encephalitis; 2. CSF characteristics suggest inflammatory response; 3. Patients whose CSF culture and/or blood culture results are bacterial infection according to traditional etiological examination and conform to clinical manifestations; 4. Traditional etiological examination was negative, but clinical manifestations and CSF manifestations were typical, and CSF characteristics were consistent with at least one of the following: ①CSF glucose < 1.9 mmol/L; ②CSF glucose/serum glucose < 0.23; ③CSF protein > 2.2 g/L;④CSF white blood cell count > 2000/μL or CSF multiple nuclear cell count > 1180/μL [13].

*Exclusion criteria*: 1. Patients with incomplete data; 2. Patients with tuberculosis, virus, fungus and other types of infection were considered; 3. Nosocomial infection, craniocerebral postoperative infection, tumor, autoimmune encephalitis, rheumatic immune disease and other conditions are excluded. 4. Blood culture results were negative, but refuse to accept lumbar puncture or have contraindications for lumbar puncture; 5. Pregnant patients are excluded.

### Related concepts and definitions

(I) *Confirmed cases* in this study include etiological confirmed cases (simultaneously meeting inclusion criteria 1, 2, and 3) and clinical confirmed cases (simultaneously meeting inclusion criteria 1, 2, and 4). (II) *Positive rate* refers to the proportion of the number of positive tests (including true positive and false positive) to the number of tests; *Detection rate* refers to the proportion of the number of people who are positive and the result is consistent with the clinical diagnosis (true positive) to the number of people tested.

### mNGS quality control

The sequencing depth of the CSF mNGS data is usually 20 M. Considering the difference in the microbial genome, the detected sequences are first normalized to reads per million (RPM), then the ratio of the RPM of clinical to the negative quality control (NTC) was calculated. When RPM-r10, the report result is positive [5].

### Statistical analysis

SPSS 23.0 was used to statistically analyze the data. Measurement data were expressed as median (interquartile range, IQR) or mean ± standard deviation(SD), counting data were expressed as rate, and rank sum test, and chi-square test or Fisher's exact probability method were used for comparison between groups, and *P* < 0.05 was considered statistically significant.

## Results

### Clinical features of 63 patients were shown in Table 1

**Table 1:** Clinical features of 63 patients

Characteristic	Value
Age-Mean ± SD	49.49 ± 14.97
Number of days in hospital – M(IQR)	22 (20)
Male: female	44:19
Disturbance of consciousness: Yes/No	41:22
Abnormal brain imaging: Yes/No	30:33
Meningeal stimulation Sign: Yes/No	41:22
Airway support: Yes/No	17:46
Combined basic disease: Yes/No	45:18
Combined acute stroke: Yes/No	4:59
Combined otogenic infection or nasal infection: Yes/No	10:53
CSF culture/Blood culture/CSF mNGS cases	56/46/44
The number of cases performed by all three methods	29

#### Summary of pathogens detected in 63 patients

**Fig. 2:**
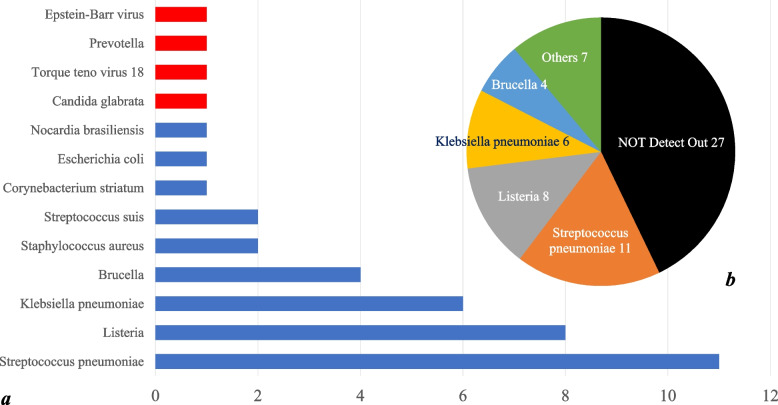
**a** Summary of pathogens detected by 3 methods in 63 patients. The abscissa represents the number of cases, the ordinate represents the type of pathogens detected, the red part indicates that the detection results are inconsistent with the clinical diagnosis (All four false-positive pathogens were detected by NGS), and the blue part indicates that the detection results are consistent with the clinical diagnosis. **b** Pie chart of pathogenic bacteria detected in 63 patients. Others include Staphylococcus aureus (2 cases), Streptococcus suis (2 cases), Nocardia brasiliensis (1 case), Candida glabrata (1 cases) and Corynebacterium striatum (1 case)

Forty-four of the 63 patients completed CSF mNGS, and a total of 13 pathogens were identified. Among the 13 pathogens, 9 were true pathogenic bacteria, and 4 were polluting microorganisms. As traditional methods do not additionally identify other pathogens, the true pathogenic bacteria detected by mNGS also represents the pathogenic bacteria detected the pathogens detected by all methods.2 samples were both infected with two pathogens (Nocardia brasiliensis combined with Torque teno virus 18, Klebsiella pneumoniae combined with Candida glabria). However, combined with clinical manifestations and CSF characteristics of patients, among them, Torque teno virus 18 and Candida glabrata were interpreted as false positive, Nocardia brasiliensis and Klebsiella pneumoniae were interpreted as pathogenic bacteria, the results of 2 samples were ultimately interpreted as false positive (1 case was Timon Prevos and 1 case was Epstein-Barr virus), the remaining results were interpreted as true positive, and the pathogenic bacteria detected mainly were Streptococcus pneumoniae (11 cases) and Listeria glabella (8 cases). Klebsiella pneumoniae (6 cases), Brucella (4 cases), Staphylococcus aureus (2 cases), Streptococcus suis (2 cases). All pathogens detected are shown in Fig. 2a, and the distribution of pathogens detected as pathogenic bacteria is shown in Fig. 2b.

### Group result

**Fig. 3:**
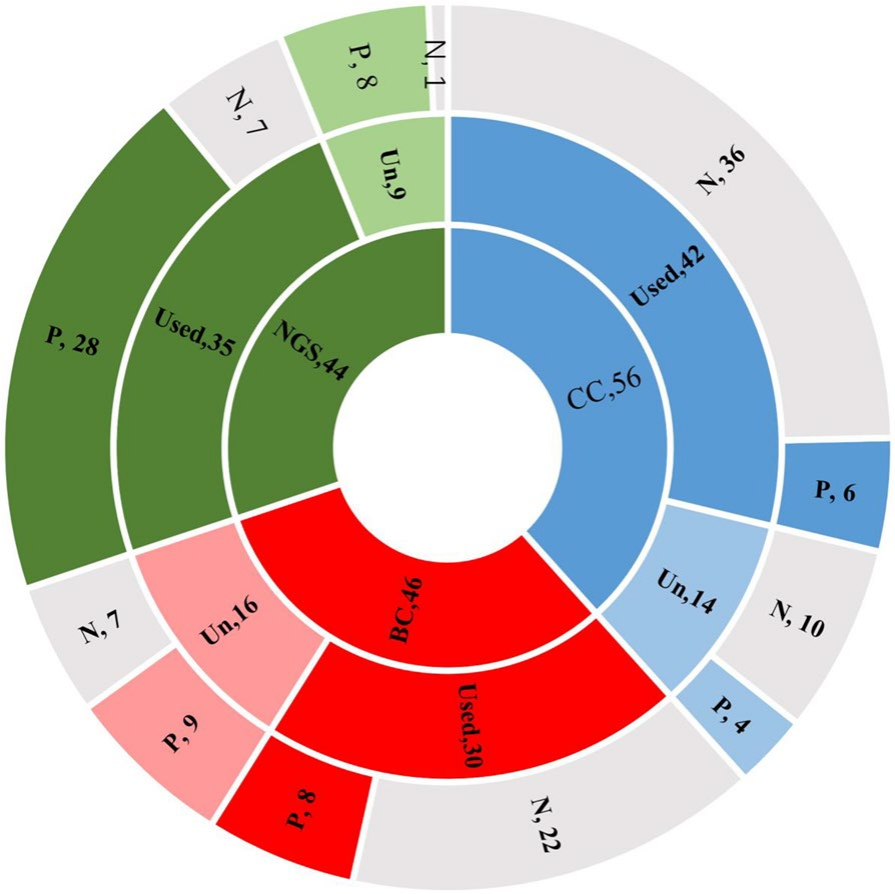
The number of patients in three groups and subgroups, and the distribution of positive and negative patients detected within each subgroup. CC: CSF culture; BC: blood culture; Un: antibiotics unused before sampling; Used: antibiotics used before sampling; P: positive; N: negative

Depending on the detection method, patients were divided into the CSF culture (CC) group(56cases), blood culture (BC) group(46cases) and CSF mNGS group (44cases), shown in the inside ring. The subgroups were divided according to whether antibiotics were used before sampling, shown in the middle ring. Light colors indicate antibiotics unused (Un) and dark colors indicate antibiotics already used (Used) before sampling. The outside ring shows number of positive and negative result. Color means positive(P), and gray means negative(N). The specific results above are shown in Fig. 3. The baseline data of blood and CSF in three groups are shown in the Table 2.

**Table 2:** Baseline data of the three groups

Groups	Cases	Blood				CSF		Glu Ratio
		WBC (*10^9^/L)	NEUT (*10^9^/L)	CRP (mg/L)	PCT (ug/L)	WBC (*10^6^/L)	Pro (g/L)	
CSF culture	56	13.19 (11.60)	11.14 (11.10)	103.57 (178.58)	1.25 (5.24)	895 (1775)	2.71 (2.42)	0.23 (0.29)
Blood culture	46	13.50 (10.45)	11.74 (10.25)	109.25 (177.73)	2.96 (5.48)	895 (1503)	2.77 (2.35)	0.16 (0.33)
CSF mNGS	44	11.70 (10.73)	10.31 (10.83)	78.12 (177.43)	1.04 (4.41)	800 (1360)	2.65 (2.59)	0.26 (0.29)

Glu Ratio means the ratio of CSF glucose to serum glucose. No statistically significant differences in the above three groups

### Comparison between the detection of CSF mNGS and traditional methods

**Fig. 4:**
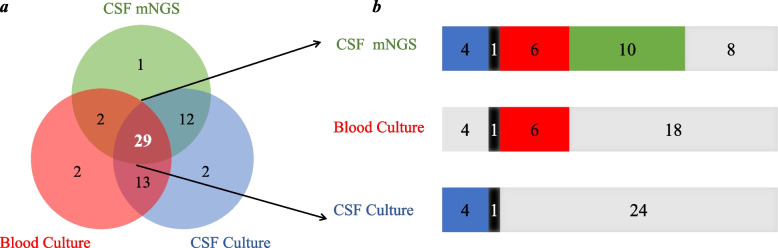
**a** Wayne diagram of 63 patients with different detection methods. **b** A total of 29 of the 63 patients completed the three methods. Gray means not detected, colored means detected; Blue is the co-detection of CSF culture and CSF mNGS, black is the co-detection of three methods, red is the co-detection of CSF mNGS and blood culture, green is the detection of CSF mNGS alone

Among the 63 patients, 29 patients completed all three methods, as shown in Fig. 4a and b shows distribution of detected cases using the three methods.

### Comparison of CSF mNGS with traditional methods after antibiotic intervention

**Fig. 5:**
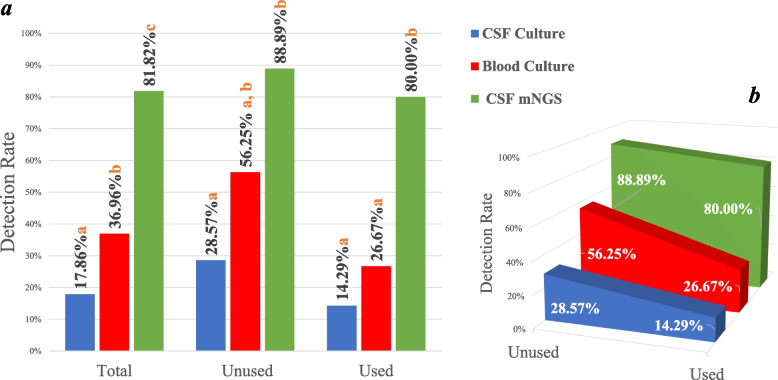
**a** Comparison of detection rate of three methods. Unused: antibiotics unused before sampling; used: antibiotics used before sampling; Three methods a, b and c have statistical difference. **a**, **b** and **c** are statistically different in pairs, and there is no statistical difference when the logo letters are the same. **b** Comparison of the detection rate of antibiotic use among the three methods. P values of CSF culture, blood culture and mNGS were 0.247, 0.06, 1, respectively

Of all 63 patients, 56 underwent CSF culture, of which 10 were detected and all were true positive; 46 underwent blood culture, of which 17 were detected and all were true positive; 44 underwent CSF mNGS, of which 36 were true positive and 2 were false positive. Two additional pathogenic microorganisms were found in 36 true positive cases, which were finally interpreted as polluting microorganisms.. The overall detection rate of each group was calculated, and the three groups were further divided into 2 subgroups according to whether antibiotics were used before sampling. The detection rates of each subgroup were compared and statistically analyzed, and the results are shown in Fig. 5a. The trend of detection rate after antibiotic use was observed, and the results are shown in Fig. 5b.

### How long antibiotics are used before sampling may affect the detection rate?

**Fig. 6:**
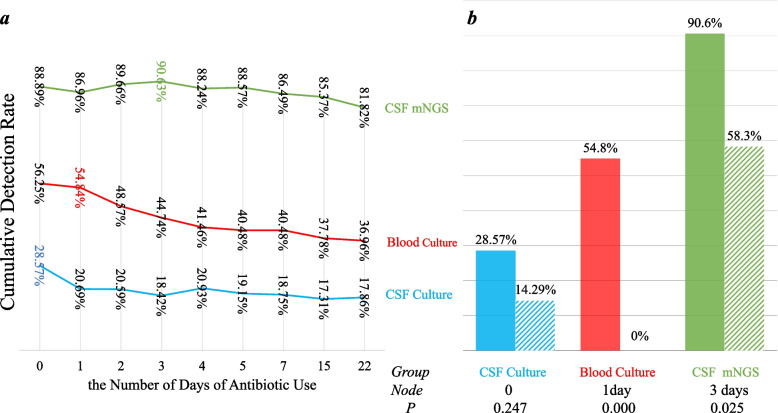
**a** Downtrend chart of the cumulative detection rate of the three methods with the number of days of antibiotic use. **b** Comparison of the cumulative detection rates before and after time nodes of the three methods

Calculate the cumulative detection rate of each time node (in days) of each group, that is, the detection rate of antibiotics used several days before sampling, make a line chart as shown in Fig. 6a. 0 represents all patients who did not use antibiotics, 1 represents patients who did not use antibiotics and patients who used antibiotics within one day before sampling, 2 represents patients who did not use antibiotics and patients who used antibiotics within two days before sampling, and so on. Take the day with the fastest line decline as the time node, and statistically analyze the detection rate before and after the node, and the results are shown in Fig. 6b. The results showed that the detection rate decreased after antibiotic use in the CSF culture group, but there was no significant difference (*p* = 0.247). The detection rate decreased significantly after 1 day of antibiotic use in the blood culture group (*p* = 0.000) and in the CSF mNGS group after 3 days of antibiotic use (*p* = 0.025).

## Discussion

CNS infectious diseases, especially purulent meningitis, are rapidly progressive and fatal diseases that neurologists often have to face. Timely and correct diagnosis is the premise of effective treatment. The traditional diagnostic paradigm is often based on positive diagnosis of CSF smear, culture or blood culture, not only the detection rate is low but the time delay. Therefore, how to detect pathogens quickly and efficiently is still an urgent problem to be solved [14]. Emerging mNGS has advantages in terms of timeliness, detection rate, and identification of pathogens in cases of unexplained infection [15]. A total of 29 of 63 patients in this study completed all three methods (Fig. 4a). In the 29 patients, CSF mNGS detected 10 more cases than the traditional methods, while the traditional methods did not detect more than CSF mNGS (Fig. 4b), confirming that the CSF mNGS detection ability was better than the traditional methods. The comparison of the detection rates of the three methods in this paper also confirms again that mNGS could not only show the pathogen more intuitively and specifically, but also have a higher detection rate than the traditional methods. In the real world, due to the rapid onset and high fatality rate of purulent meningitis, patients have been exposed to different kinds of antibiotics in the process of referrals from primary hospitals. So, the characteristics of CSF are often atypical, CSF culture is often negative [16], and diagnosis is often in a deadlock [17, 18]. For these patients, clinicians are eager to know whether CSF mNGS has advantages over traditional methods. In this study, when analyzing the effect of antibiotics on the detection of mNGS and traditional methods, the nodes of antibiotic use were not limited in order to more truly present the status of patients at the time of visit. We found that the detection rate of CSF mNGS of 80.00% (28/35) was still higher than that of CSF culture of 14.29% (6/42) and blood culture detection rate of 26.67% (8/30) after antibiotics, suggesting that mNGS may be a good supplement to traditional methods. The detection rate of the mNGS group in this study was higher than that of previously published mNGS [5, 14, 19], which was related to the fact that the population enrolled in the study themselves were confirmed cases with purulent meningitis, and the population did not involve pathogens with low detection rates of Mycobacterium tuberculosis and viruses.

In further analysis of each subgroup, when antibiotics were not used before sampling, the detection rates of blood culture and mNGS were comparable, and both were higher than that of CSF culture. However, after antibiotic use, the detection rate of the blood culture group was significantly reduced and the detection rate of mNGS was significantly higher than that of the two traditional methods, indicating that the blood culture results were susceptible to antibiotics, but the detection rate of mNGS was less affected by antibiotics, that is, the detection rate before and after antibiotics was relatively stable, which is consistent with previous findings [20]. Looking through the relevant data, it was found that the advantage of mNGS diagnosis was related to the detection principle of this method. The positive results of CSF and blood culture depended on the number of viable bacteria, and the detection rate can be affected by bacterial lysis after antibiotic administration. While mNGS only needs to identify trace amounts of microbial DNA fragments, so pathogens still have a high detection rate after antibiotic treatment [4]. In our study, the detection rate of blood culture is slightly lower than mNGS, but still higher than CSF culture, may be based on the following reasons: most intracranial infections are blood-borne infection, a few are nasal or ear infections. Due to the blood–brain barrier, the number of pathogens in the blood significantly exceeds that in the CSF, so the detection rate of blood culture is relatively high, and after antibiotics enter the blood, the pathogens quickly dissolve, and the detection rate quickly decreases. In recent years, studies on the protection mechanism of blood–brain barrier have been constantly updated [21–24]. In addition, we also studied the effect of antibiotics on each method, suggesting that the detection rate after antibiotic use decreased in the three groups, but there was no statistical difference. Specifically, the detection rate of CSF mNGS decreased slightly after antibiotic use, while the blood culture group and the CSF group showed a significant downward trend. Considering the small number of patient samples in this study, the next step can expand the sample to improve this demonstration.

Although the detection rate of CSF mNGS is high, it is also affected by the length of antibiotic intervention before sampling. Studies [12] have indicated that the detection rate of mNGS is also affected when the empirical anti-infection treatment is given more than 4 days. Our study also counted the decreasing trend of detection rate with antibiotic use (Fig. 6a, b). The results showed that, compared with the traditional methods, the detection rate of the mNGS group decreased significantly after 3 days of antibiotic use, suggesting that the use of mNGS in patients with purulent meningitis during this period has more advantages. For the cases with positive mNGS result after 3 days of antibiotic use, due to the small number of cases, we cannot again affirm whether the positive detection is related to the low immunity, high load of bacteria or the pathogen itself is not easy to be killed. In the future, we can make specific analysis when the number of cases is large.

## Limitations

This retrospective study also has some shortcomings: 1. Not all cases have completed the initial CSF culture and blood culture according to the standard diagnostic procedures. Of course, because of this, this result can more truly reflect the diversity and complexity of clinical diagnosis and treatment process, as well as the advantages of the CSF mNGS in the diagnosis and treatment of such cases. 2. According to the diagnostic criteria referred to in this paper, a part of patients with negative etiology and CSF traits that do not meet the clinical diagnostic criteria were missed, so it is not possible to evaluate whether CSF mNGS has an advantage after antibiotic use in this population.

## Conclusions

Currently, CSF mNGS has become an important and practical technique for the identification of the etiology of CNS infection. For patients who have already used antibiotics, the detection rate of mNGS is significantly better than that of traditional methods, which may be the direction for accurate etiological detection of CNS infection in the future. However, as the detection rate of mNGS decreases with the extension of antibiotic use, we still need to send samples for examination as early as possible.

## Data Availability

The raw reads including whole-genome resequencing in this study are publicly available at the NCBI Sequence Read Archive under accession code PRJNA996497. The sequence reads of 44 download variety accessions are available from the website http://www.ncbi.nlm.nih.gov/bioproject/996497.

## Abbreviations

mNGS: Metagenomic next-generation sequencing
CSF: Cerebrospinal fluid
RPM: Reads per million
NTC: Negative quality control
CNS: Central nervous system
